# Feasibility and acceptance of transdermal auricular vagus nerve stimulation using a TENS device in females suffering from long COVID fatigue

**DOI:** 10.1007/s00508-025-02501-1

**Published:** 2025-02-19

**Authors:** Veronika Pfoser-Poschacher, Mohammad Keilani, Margarete Steiner, Jim Schmeckenbecher, Ralf Harun Zwick, Richard Crevenna

**Affiliations:** 1 Department of Physical Medicine, Rehabilitation and Occupational Medicine, Medical University of Vienna, Waehringer Guertel 18–20, 1090 Vienna, Austria; 2 Department of Psychoanalysis and Psychotherapy, Medical University of Vienna, Vienna, Austria; 3 Center for Mental Health, Clinic for Addiction Medicine and Dependent Behavior, Klinikum Stuttgart, Stuttgart, Germany; 4 Therme Wien Med, Ludwig Boltzmann Institut für Rehabilitationsforschung, Kurbadstr. 14, 1100 Vienna, Austria

**Keywords:** Transcutaneous auricular vagus stimulation, TENS, Long COVID, Parasympathetic

## Abstract

**Background:**

The COVID-19 pandemic has led to significant health challenges, with some individuals developing long COVID characterized by persistent symptoms such as fatigue, dyspnea and cognitive difficulties lasting weeks or months after infection. This condition predominantly affects women and may involve prolonged inflammation and autonomic nervous system dysfunction. Current treatments focus on symptom relief and vagus nerve stimulation (VNS) is being investigated for its potential therapeutic benefits.

**Methods:**

This pilot study was a prospective, blinded, randomized controlled trial involving 36 female long COVID patients aged 18–70 years. Participants were assigned to three groups receiving VNS at frequencies of 10 Hz, 25 Hz, or a control of 2 Hz for 3 months. Outcomes were assessed at baseline, after 4 and 12 weeks.

**Results:**

The study revealed that all VNS treatment groups experienced reduction in symptoms associated with long COVID, particularly in fatigue and dyspnea, after 12 weeks. Participants across all frequencies reported an improvement in health-related quality of life. Heart rate variability remained stable throughout the trial, and no significant changes in morning salivary cortisol levels were seen across groups.

**Discussion:**

Vagus nerve stimulation may offer therapeutic benefits for women with long COVID, particularly in reducing fatigue and dyspnea. The treatment was found to be safe, with no significant side effects reported; however, further research with larger study groups is needed to confirm these findings and examine the long-term effects of VNS on autonomic nervous system function.

## Introduction

Infections with SARS-CoV‑2 (COVID-19) have become a global health crisis, causing high morbidity and mortality rates. While around 80% of those infected experience mild to moderate symptoms, about 5% develop critical conditions [1]. A small percentage of patients recovering from COVID-19 develop persistent or new symptoms lasting for weeks or months, referred to as long COVID [2, 3]. Long COVID is more prevalent in women than men [4] and is characterized by ongoing or new symptoms occurring more than 4 weeks after the initial infection. Most patients with long COVID show negative PCR tests, indicating microbiological recovery [1].

Long COVID can be divided into two stages based on symptom duration. If symptoms persist for more than 4 but fewer than 12 weeks, it is classified as ongoing or subacute COVID-19. Symptoms lasting beyond 12 weeks are referred to as post-COVID [1–3, 5]. The range of symptoms is wide and includes fatigue, persistent smell or taste disturbances, sleep problems, dyspnea, palpitations and tachycardia. Other symptoms include chronic cough, chest pain, joint and muscle pain, sensory disturbances, headaches, dizziness, and orthostatic dysfunction such as postural orthostatic tachycardia syndrome (POTS) [1–3]. Cognitive issues, anxiety, depression, hair loss and intermittent fever are also common [1–3].

The pathophysiology of long COVID remains unclear; however, persistent symptoms may be linked to prolonged inflammation and immune dysregulation following the infection, possibly driven by a cytokine storm [6]. There is evidence that COVID-19 may affect the autonomic nervous system (ANS), causing dysfunction [6]. Survivors of COVID-19 also appear to be at increased risk for posttraumatic stress disorder [7].

Dysautonomia or ANS dysfunction, involves imbalances in sympathetic or parasympathetic activity. The exact mechanisms are complex, with uncertainty about whether these are virus-induced, autoimmune or secondary changes. Increased sympathetic activation may contribute to a cytokine storm, while vagus nerve stimulation (VNS) has potential anti-inflammatory effects through acetylcholine receptors [8]. Symptoms of dysautonomia include fatigue, orthostatic hypotension, POTS, and abnormal heart rate variability (HRV) [6, 9].

Studies suggest that long COVID may lead to vagus nerve atrophy, indicating parasympathetic involvement and prolonged sympathetic skin response (SSR) latency, suggesting sympathetic dysfunction [10]. Additionally, long COVID may be associated with reduced HRV [9, 11].

Currently, treatment for long COVID remains symptomatic. Activation of the vagus nerve may reduce inflammation through the vagal anti-inflammatory cholinergic pathway, which inhibits proinflammatory cytokines such as TNF, IL‑6, and IL-1beta [12–15]. Transcutaneous electrical nerve stimulation (TENS), including transcutaneous auricular vagus nerve stimulation (taVNS), has been proposed as a potential treatment. Frequencies of 10–30 Hz can stimulate vagal afferents, while lower frequencies (2–4 Hz) are used for treating polyneuropathy [16, 17].

The aim of the present pilot study is to investigate the acceptability, feasibility, and implementation of a 3-month VNS treatment with two different frequencies (10 Hz, 25 Hz) in female long COVID patients, along with a control frequency (2 Hz) [16, 18]. Additionally, the study seeks to describe the effects on autonomic nervous system parameters as well as long COVID symptoms through a pre/post comparison.

## Material and methods

### Trial design and overview

This pilot study was a prospective, blinded, randomized controlled trial (RCT) designed to investigate the acceptability, feasibility and effect of vagus nerve stimulation (VNS) in long COVID patients. A total of 36 female participants, aged 18–70 years, with a confirmed diagnosis of long COVID, were enrolled. Participants were randomly assigned to one of three groups: 12 patients received VNS at 10 Hz, another 12 at 25 Hz, and the remaining 12 formed a control group with VNS at 2 Hz. The patients were blinded to their group assignments throughout the trial.

Recruitment began on 3 March 2023 and the last patient was completed on 30 September 2024. Inclusion criteria included women aged 18–70 years with a confirmed diagnosis of long COVID, based on medical history, physical examination, and relevant findings, who provided signed informed consent. Exclusion criteria encompassed individuals with cochlear implants, a surgically severed vagus nerve, systemic diseases (e.g., malignancies, autoimmune disorders), orthopedic, rheumatological or neurological diseases, postoperative conditions, recent ear injuries or infections, psychiatric disorders, fever, pacemakers, implanted cardioverter defibrillators (ICD), history of ear surgery, seizure disorders, Meniere’s disease, negative experiences with electrotherapy, or insufficient German language skills.

### Intervention

The intervention consisted of a 3-month home treatment using transcutaneous electrical nerve stimulation (TENSeco®, CE197, Schwa-Medico GmbH, Ehringshausen, Germany), using an ear electrode (3 DTS DISPOSITIF MEDICAL, Rouffach, France) for daily VNS sessions. Stimulation was applied to the left ear for 30 min each evening, using biphasic waveforms. The three groups received different stimulation frequencies: 10 Hz, 25 Hz, and 2 Hz (control group).

Participants received instructions on how to use the device during an initial clinic visit. The VNS was performed daily, and outcomes were assessed at baseline (T0), after 4 weeks (T0 + 4 weeks), and at the end of the 12-week period (T0 + 12 weeks).

### Outcomes

The present pilot study focused on the acceptability, feasibility and adherence to VNS treatment. This included evaluating the duration and frequency of VNS sessions, reasons for adherence or nonadherence and participants’ subjective perceptions of the treatment effects. Feasibility and acceptance were assessed by using the Austrian school grading system (1: very good, 2: good, 3: satisfactory, 4: sufficient, 5: not sufficient).

Further outcomes targeted the impact of VNS on the autonomic nervous system and long COVID symptoms. Heart rate variability (HRV) was assessed with parameters, such as SDNN and RMSSD recorded using the GE Healthcare SEER 1000 Holter recorder (Beer Medizintechnik GmbH, Gleisdorf, Austria). Heart rate, blood pressure, and rate pressure product were measured by using the Boso Medicus device (BOSCH + SOHN GmbH, Jungingen, Germany). Salivary cortisol levels were collected at 8 a.m. and analyzed in a clinical laboratory. Fatigue levels were assessed with the Brief Fatigue Inventory (BFI). Health-related quality of life (HRQOL) was measured using the SF-36 questionnaire. Dyspnea severity was evaluated using the modified Borg scale, and sleep quality was assessed using the Insomnia Severity Index (ISI). Adverse events were monitored during all follow-up assessments.

### Statistical analysis

Data were analyzed using descriptive statistics, focusing on intragroup and intergroup comparisons at the three time points: T0 (baseline), T0 + 4 weeks, and T0 + 12 weeks (end of treatment). Mean, standard deviation, median and interquartile range were calculated for continuous variables. For normally distributed variables, the mean and standard deviation were used, while for non-normally distributed variables, the median and interquartile range were reported. Pre-intervention and post-intervention comparisons were conducted within and between groups. All statistical analyses were carried out using SPSS (IBM Corp., Armonk, New York, United States) and R software (R Foundation, Indianapolis, Indiana, United States). As this was a pilot study, descriptive statistics were used, and the results were exploratory in nature.

## Results

### Participants

Out of 44 healthy young female participants assessed for eligibility, 36 met the criteria for inclusion in the study. After randomization, 1 participant dropped out, resulting in 35 women who completed the trial. The participants were all Europeans, aged between 24 and 68 years, with an average age of 43 ± 10 years. The body mass index (BMI) ranged from 17.3 to 34.6 kg/m^2^, with a mean BMI of 23.9 ± 4.1 kg/m^2^ (Table 1).

**Table 1:** Demographic data

		10 Hz (*N* = 12)	25 Hz (*N* = 11)	2 Hz (*N* = 12)
*Age *(years)	Mean (SD)	45 (± 7)	41 (± 13)	44 (± 10)
	Distribution no. (%)			
	18–29 years	0 (0)	2 (18)	0 (0)
	30–39 years	3 (25)	4 (37)	5 (42)
	40–49 years	3 (25)	2 (18)	4 (33)
	50–59 years	6 (50)	2 (18)	2 (17)
	60–70 years	0 (0)	1 (9)	1 (8)
*Weight *(kg)	Mean (SD)	69.83 (± 12)	66.36 (± 15.3)	64.67 (± 10.6)
*BMI *(kg/m^2^)	Mean (SD)	24.5 (± 4.3)	23.7 (± 5.1)	23.4 (± 3.3)
	Distribution no. (%)			
	< 18.5	1 (8)	0 (0)	0 (0)
	18.5–24.9	5 (42)	8 (73)	7 (58)
	25–29.9	4 (33)	1 (9)	5 (42)
	30–34.9	2 (17)	2 (18)	0 (0)
*Height* (m)	Mean (SD)	1.69 (± 0.06)	1.67 (± 0.04)	1.66 (± 0.05)

*SD* standard deviation, *BMI* body mass index

### Clinical outcomes

The median device usage time across all participants was 2267 min (IQR 964), with individual frequencies showing slight variation (10 Hz: 2325.5 min, 25 Hz: 2180.5 min, 2 Hz: 2258.5 min). The number of uses varied between groups, with a median of 81 sessions across all participants (IQR 36). The median intensity of VNS was recorded at 9.5 mAe (IQR 6.5) in the whole population with a range 1–31 mA.

The average daily treatment time was 30 min (IQR 0) across all groups. Subjective numeric ratings on a 1–5 scale (where 1 means VNS is excellent and 5 means poor) revealed a score of 3 (IQR 1.25) after 4 weeks and improved to a score of 2 (IQR 2.12) after 12 weeks. Notably, the most significant improvement was observed in the 10 Hz group, which experienced a decrease of 0.5 points, compared to a decrease of 0.25 points in the 25 Hz group and an increase of 0.75 points in the 2 Hz group (Table 2).

**Table 2:** VNS application metrics

		Total (*N* = 35)	10 Hz (*N* = 12)	25 Hz (*N* = 11)	2 Hz (*N* = 12)
*Duration of application (min)*	Median (IQR)	2267 (964)	2325.5 (1061)	2180.5 (1334.25)	2258.5 (359.25)
	Min–max	21–6764	935–6764	21–2701	1039–2649
*Intensity (mA)*	Median (IQR)	9.5 (6.5)	9 (5)	9.5 (5.75)	10.5 (9.25)
	Min–max	1–31	1–31	5–20	2–16
*Number of applications*	Median (IQR)	81 (36)	84 (38.75)	76 (47.75)	83 (11.5)
	Min–max	3–249	36–249	3–91	38–96

*IQR* interquartile range

Of the participants 72% found VNS to be effective, 14% consider it ineffective and another 14% were unsure.

Heart rate variability (HRV), assessed through standard deviation of NN intervals (SDNN) and root mean square of successive differences (RMSSD), indicated moderate HRV among participants. The SDNN values showed stability over time for the whole group, with a mean of 59 ms at baseline, decreasing slightly after 4 weeks (54 ms) and returning to 59 ms after 12 weeks, while median RMSSD values decreased slightly from 38 ms at baseline to 37 ms after 12 weeks. Typical reference values for SDNN in healthy individuals range from 50 to 150 ms, while RMSSD values generally lie between 20 and 50 ms [19].

The mean heart rate pressure product (HRPP) remained within normal limits, showing a baseline mean of 8827 and increasing to 9255 after 12 weeks. Normal values range from 7000 to 12,000 in healthy individuals.

Values at the lower end of the range indicate a lower cardiac workload, while higher values within this range still fall within normal limits [20].

Similarly, pulse rates were within normal ranges, averaging 75 bpm (beats per min) at baseline and rising to 77 bpm at the 12-week follow-up. The resting heart rate ranges in healthy adults from 60 to100 bpm [21].

Morning salivary cortisol levels also fell within the normal range, with a median of 0.247 µg/dl at baseline and 0.29 µg/dl after 12 weeks. The standard reference value for morning salivary cortisol (between 6:00am and 10:00 am) is under 0.783 µg/dl, according to the laboratory guidelines of the Department of Laboratory Medicine, Vienna General Hospital, Medical University of Vienna.

The post-COVID-19 functional status scale (PCFS) demonstrated an improvement, decreasing from a median score of 3 points at baseline to 2 points at the 12-week assessment.

Dyspnea severity, evaluated using the modified Borg scale, showed a decrease from a median score of 3 points at baseline to a median of 2 points after 12 weeks.

Fatigue levels, measured by the Brief Fatigue Inventory (BFI), revealed a reduction with mean BFI scores decreasing from 5.5 points at baseline to 4.5 points after 12 weeks.

Sleep quality, assessed with the ISI, showed improvements from 14 points at baseline to 11 points after 12 weeks.

Health-related quality of life (HRQOL) was evaluated using the SF-36 questionnaire. The physical component summary improved from 32 to 36 points over the 12-week period, while the mental component summary showed an increase from 40 to 45 points (Table 3).

**Table 3:** Outcome parameters

		Total (*N* = 35)	10 Hz (*N* = 12)	25 Hz (*N* = 11)	2 Hz (*N* = 12)
*SDNN*	Baseline	59 (± 20)	60 (± 22)	57 (± 24)	59 (± 11)
Mean (SD); ms	+4 weeks	54 (± 20)	58 (± 18)	52 (± 25)	53 (± 18)
	+12 weeks	59 (± 22)	63 (± 21)	56 (± 23)	57 (± 24)
*RMSSD*	Baseline	38 (± 25)	38 (27)	37 (21)	41 (29)
Median (IQR); ms	+4 weeks	33 (± 28)	33 (39)	26 (21)	35 (33)
	+12 weeks	37 (± 24)	41 (22)	39 (20)	27 (33)
*HRPP*	Baseline	8828 (± 2079)	8867 (± 1936)	8606 (± 1760)	9106 (± 2624)
Mean (SD); absolute no.	+4 weeks	8827 (± 1855)	8140 (± 1419)	9108 (± 1684)	9365 (± 2301)
	+12 weeks	9255 (± 2277)	9093 (± 1924)	8946 (± 2331)	9334 (± 2449)
*Pulse*	Baseline	75 (± 11)	74 (± 10)	74 (± 10)	77 (± 13)
Mean (SD); bpm	+4 weeks	75 (± 9)	73 (± 8)	78 (± 9)	76 (± 9)
	+12 weeks	77 (± 10)	77 (± 11)	77 (± 12)	76 (± 8)
*Cortisol*	Baseline	0.247 (0.207)	0.243 (0.152)	0.37 (0.34)	0.259 (0.175)
Median (IQR); µg/dl	+4 weeks	0.284 (0.261)	0.266 (0.18)	0.346 (0.246)	0.266 (0.266)
	+12 weeks	0.284 (0.21)	0.302 (0.105)	0.252 (0.418)	0.284 (0.169)
*PCFS*	Baseline	2.8 (± 0.7)	2.8 (± 0.6)	2.8 (± 0.6)	2.8 (± 0.8)
Mean (SD); absolute no.	+4 weeks	2.5 (± 0.7)	2.5 (± 0.8)	2.6 (± 0.7)	2.3 (± 0.8)
	+12 weeks	2.4 (± 0.8)	2.3 (± 0.8)	2.3 (± 0.8)	2.4 (± 0.8)
*BORG*	Baseline	3 (2.25)	3 (2.25)	3.25 (2)	3 (2.5)
Median (IQR); absolute no.	+4 weeks	2 (2)	2 (1.25)	2 (3.25)	2 (3)
	+12 weeks	2 (2)	1.5 (1)	2.5 (1.25)	2 (1.75)
*BFI*	Baseline	5.5 (± 1.7)	5.4 (± 4.5)	6.2 (± 1.6)	4.9 (± 1.4)
Mean (SD); absolute no.	+4 weeks	4.8 (± 1.9)	4.5 (± 2.1)	5.4 (± 1.9)	4.6 (± 1.8)
	+12 weeks	4.5 (± 2.2)	3.7 (± 2.6)	5.4 (± 1.8)	4.5 (± 2.1)
*ISI*	Baseline	13.7 (± 5.5)	10.3 (± 6.6)	16.8 (± 3.3)	14.2 (± 4.4)
Mean (SD); absolute no.	+4 weeks	12.3 (± 4.3)	10.6 (± 4.7)	13.5 (± 3.6)	12.7 (± 4.2)
	+12 weeks	11.1 (± 6.2)	8.3 (± 6.1)	14.3 (± 5)	10.8 (± 6.3)
*PCS*	Baseline	32 (± 9)	30 (± 6)	34 (± 13)	33 (± 8)
Mean (SD); absolute no.	+4 weeks	34 (± 10)	33 (± 9)	33 (± 12)	37 (± 9)
	+12 weeks	36 (± 10)	38 (± 10)	36 (± 10)	35 (± 10)
*MCS*	Baseline	40 (± 11)	45 (± 11)	34 (± 11)	42 (± 9)
Mean (SD); absolute no.	+4 weeks	43 (± 10)	49 (± 9)	41 (± 10)	40 (± 11)
	+12 weeks	45 (± 11)	51 (± 7)	40 (± 13)	44 (± 10)

*SDNN* standard deviation of the NN intervall, *RMSSD* root mean square of successive differences, *HRPP* heart rate pressure product, *PCFS* post-COVID-19 functional status, *SD* standard deviation, BORG Borg scale, *ISI* insomnia severity index, *bpm* beats per minute, *BFI* brief fatigue inventory, *PCS* physical component summary of the short-form 36 health survey, *MCS* mental component summary of the short-form 36 health survey

### Adverse events

No suspected unexpected serious adverse reactions (SUSAR) or serious adverse events (SAE) were reported. Patients noted side effects, including tinnitus, difficulty falling asleep, restless legs syndrome, occasional ear pain, pulsating sensations in the ear area, itching, headaches, tingling, twitching, and sleep disturbances (Table 4).

**Table 4:** Summary of Adverse Events

		10 Hz (*N* = 12)	25 Hz (*N* = 11)	2 Hz (*N* = 12)
*Any AE*	No. of participants (%)	4 (33)	3 (27)	5 (42)
*AE reported in ≥* *1% of participants in either treatment group*	No. of participants (%)			
	Insomnia (onset/maintenance)	2 (17)	0 (0)	1 (8)
	Itching/tingling	1 (8)	0 (0)	2 (17)
	Headache	0 (0)	2 (18)	0 (0)
	Ear pain	1 (8)	1 (9)	0 (0)
	Pulsating	0 (0)	0 (0)	1 (8)
	Restless legs	0 (0)	0 (0)	1 (8)

*AE* adverse event, *SD* standard deviation

## Discussion

This pilot study explored the acceptability, feasibility and preliminary effects of transcutaneous vagus nerve stimulation (VNS) in female patients suffering from long COVID. The results demonstrate promising trends in improving various symptoms and autonomic nervous system parameters, indicating that VNS could serve as an additional therapeutic approach for managing somelong COVID symptoms.

The high adherence rate to the VNS protocol, with a median treatment duration of 2267 min across all groups, suggests that participants found the intervention manageable and acceptable. This is crucial for the success of any long-term treatment strategy, especially in chronic conditions like long COVID, where consistent adherence is required for effective symptom management. The participants’ subjective ratings improved during the treatment, with the 10 Hz group experiencing the greatest reduction in symptom scores and the most noticeable boost in perceived well-being. This is consistent with the existing limited literature suggesting that higher frequency stimulation may provide more robust therapeutic effects [16, 18].

While the study primarily focused on symptom relief, the assessment of ANS parameters revealed stable HRV measures, with the mean SDNN remaining consistent throughout the study period. This stability suggests that VNS may help maintain autonomic balance in participants, potentially countering the dysautonomia commonly observed in long COVID patients. The slight decrease in median RMSSD values from baseline to the 12-week follow-up indicates that while overall variability in heart rate remained stable, subtle changes in parasympathetic modulation could still be occurring. These findings might support the hypothesis that VNS can positively influence autonomic activity, aiding in recovery from symptoms associated with long COVID [8, 9].

Further outcomes revealed notable improvements in fatigue, dyspnea severity, sleep quality, and health-related quality of life in all groups. The mean cores of the BFI indicated a reduction in fatigue levels, a common and debilitating symptom in long COVID patients. The reduction in dyspnea severity, as evidenced by the modified Borg scale, further highlights the potential of VNS to alleviate respiratory distress, a prevalent issue in this population. The observed improvements in sleep quality, measured through the ISI, suggest that VNS may positively influence sleep disturbances frequently reported by individuals with long COVID. These outcomes are consistent with previous research indicating that vagus nerve activation can enhance sleep quality and reduce anxiety and depressive symptoms [12, 13]. Additionally, the noninvasive nature of the applied VNS, along with its simplicity in handling and cost-effectiveness, makes it a practical intervention that can be easily integrated into a rehabilitation setting [22].

The sample size was too small to detect differences between individual groups, but the overall population demonstrated improvement with VNS.

While the study reported several side effects associated with VNS, including ear pain and difficulty falling asleep, it is noteworthy that no serious adverse events were recorded. The occurrence of mild adverse events, such as itching or headaches, suggests that VNS is generally well-tolerated by participants. Continuous monitoring and documentation of these events will be crucial for informing future studies and optimizing treatment protocols.

This pilot study has several limitations that should be acknowledged. The small sample size and lack of long-term follow-up limit the generalizability of the findings. Additionally, the study focused exclusively on female participants, which may restrict the applicability of results to the broader population. Future research should aim to include larger and more diverse cohorts, as well as investigate the long-term effects of VNS on both physical and psychological outcomes in long COVID patients. It should be noted that another limitation of the study is that long COVID symptoms may show successive improvement over time, and therefore, the time factor alone could lead to symptom improvement [23]. Additionally, it is difficult to implement a true placebo control in physical studies, meaning the 2 Hz group was not a real control group but more a subtherapeutic intervention. This complicates the interpretation of a placebo comparison. Future studies should aim to compare VNS with a standard alternative treatment to strengthen the evidence.

In conclusion, the findings of this pilot study provide preliminary data supporting the acceptability and feasibility of VNS in treating long COVID symptoms. The observed improvements in symptomatology and stable autonomic parameters warrant further investigation through larger multicenter trials to establish the efficacy and safety of VNS as a treatment option for individuals suffering from long COVID.

## Abbreviations

AE: Adverse event
ANS: Autonomic nervous system
BFI: Brief fatigue inventory
BMI: Body Mass Index
BORG: Borg Scale (modified)
COVID-19: Coronavirus Disease 2019
HRPP: Heart Rate Pressure Product
HRV: Heart Rate Variability
IL: Interleukin
ISI: Insomnia Severity Index
NICE: National Institute for Health and Care Excellence
PCR: Polymerase Chain Reaction
POTS: Postural Orthostatic Tachycardia Syndrome
RCT: Randomized Controlled Trial
RMSSD: Root Mean Square of Successive Differences
SARS-CoV‑2: Severe acute respiratory syndrome coronavirus type 2
SD: Standard Deviation
SDNN: Standard Deviation of NN intervals
SF-36: 36-Item Short Form Health Survey
SSR: Sympathetic Skin Response
taVNS: Transcutaneous Auricular Vagus Nerve Stimulation
TENS: Transcutaneous Electrical Nerve Stimulation
TNF: Tumor Necrosis Factor
VNS: Vagus Nerve Stimulation
